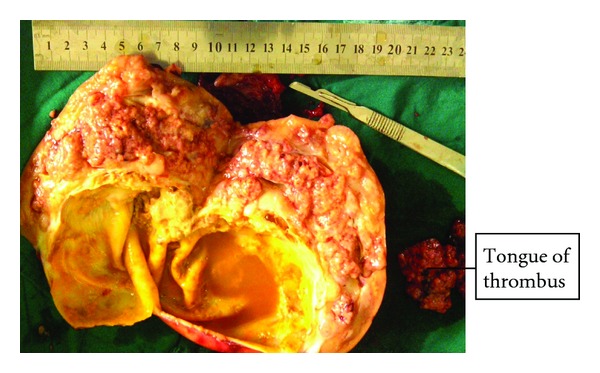# The Operative Challenges of Advanced Renal Cell Carcinoma with Vena Cava Involvement: A Report of Three Cases

**DOI:** 10.1155/2011/514373

**Published:** 2011-12-15

**Authors:** Muftau Jimoh Bioku, Abdulwaid Niran Saliu, Stephen Odunayo Ikuerowo, Olufunmilade Omisanjo, Julius Olusanmi Esho

**Affiliations:** Urology Unit, Department of Surgery, Lagos State University Teaching Hospital, Ikeja, Lagos, Nigeria

## Abstract

Surgical resection remains an important component in the care of advanced renal cell carcinoma (RCC). Some of the patients so managed had relief of symptoms and improved quality of life. However, palliative nephrectomies in late cases with vena cava involvement are not without challenges. An important factor to be considered for successful surgery is adequate vena cava management. We report in this paper three patients who had metastatic RCC. For over three decades now, researchers in Lagos had recorded the abysmal prognosis of advanced cases of RCC. Yet, late presentation and diagnosis still persisted in our environment. There is therefore the need to repackage our strategies aimed at early detection of this pathology and thus improved postoperative outcome.

## 1. Introduction

Renal cell carcinoma was first reported by Paul Grawitz in 1883. It was named after him as Grawitz tumuor, or hypernephroma, according to his belief that the tumor originated in adrenal rests at the upper pole of the kidney. Later, the origin of this tumor in renal tubular cells was documented [1].

 Accounting for 2% of all adult malignancies [2], RCC has tendency to spread into the renal vein and the IVC. Late presentation is the common initial diagnosis in our part of the world [3], making operative treatment more challenging.

In this report, we describe three cases of metastatic RCC. Each had radical nephrectomy and vena caval management. The perioperative and postoperative challenges peculiar to each case were elucidated.

## 2. Case  1

### 2.1. Clinical Presentation

A 65-year-old female was referred with seven-month history of right flank pain and weight loss. She did not have haematuria.

On examination, she was wasted. She had no pedal edema or supraclavicular lymphadenopathy. A mass was palpable in the right lumbar region extending to the right hypochondrium and crossing the midline. The mass had an irregular surface and was tender and ballotable.

**Figure 1:**
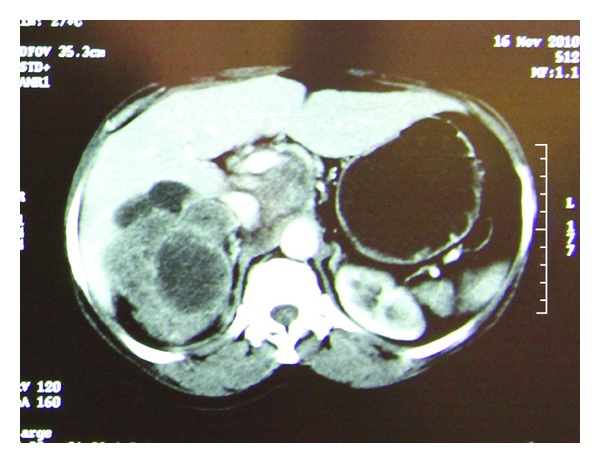


 The patient had a computerized tomography (CT) scan of abdomen and pelvis which showed a large (92 mm × 80 mm × 90 mm) lobulated mixed density renal mass arising from the upper pole and involving the ipsilateral adrenal gland. The extrarenal component indented the inferior and posterior surfaces of the liver. Enlarged lymph nodes were seen in the region of celiac axis, portocaval, and retrocaval area measuring 6 cm in largest diameter. Main renal vein on the left side revealed no definite luminal filling defect. There was narrowing of the IVC. Multiple tiny nodules were noted in the lung parenchyma bilaterally (Figure 1). 

### 2.2. Operative Procedure

Under general anesthesia, the patient was placed in the supine position with the right flank elevated with sand bag to 30° to facilitate mobilization of the tumour. The skin was prepped from nipple-line to the mid-thigh and patient draped in the usual fashion. A right transverse upper abdominal incision was made from tip of 12th rib crossing the midline 2 cm above the level of the umbilicus and extending to lateral border of the left rectus abdominis muscle. The peritoneal cavity was entered and entire abdomen explored. A firm, irregular brownish-yellow mass measuring 19 cm × 10 cm was found. This had almost completely replaced the right kidney. tumor had infiltrated the Gerota's fascia and was adherent to the inferior surface of the liver. The right renal vein and adjacent IVC wall were infiltrated. There was perihilar and para-aortic lymphadenopathy. However, there was no palpable liver metastasis.

 The colon and duodenum were free.

 Initial renal pedicle control was not possible. Thus, the tumor was mobilized inferiorly and laterally. The superior pole of the tumour was separated from the inferior hepatic surface with careful blunt dissection. The distorted renal vessels were carefully dissected, and divided in between Satinsky vascular clamps. Partial longitudinal resection of the tumor-infiltrated IVC wall was done. This was accompanied by transient hypotension which was controlled by the preinformed anaesthetists.

The ureter was then ligated and divided far beyond the level of tumour involvement. The tumour, the adrenal gland, regional lymph nodes, and resected wall of IVC were removed in one block.

The renal fossa and inferior hepatic surface were packed as the latter continued to ooze despite efforts at meticulous hemostasis. The pack was removed forty-eight hours postoperatively by the bed side.

 Patient had hypovolemic shock with acute renal failure twelve hours after-surgery. This was successfully managed in conjunction with nephrologists.

 She also had right facial palsy and bilateral pitting pedal edema up to midleg. She was discharged to the clinic ten days later.

### 2.3. Outcome and Followup

**Figure 2:**
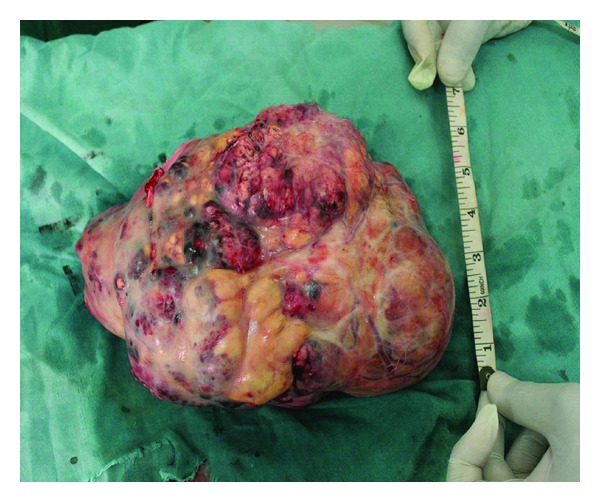


The gross pathological examination revealed a greyish-brown nodular kidney with the adrenal measuring 18.5 cm × 12 cm × 11 cm and weighing 813 g. The pelvic region showed the stump of the ureter, two ligated blood vessels measuring 1.5 cm and 1.7 cm long, respectively, with external diameter of 0.4 cm (Figure 2).

 Microscopically, the specimen showed renal tissue with large sheets of neoplastic epithelial cells separated by delicate vascular septae. These cells exhibited clear to eosinophilic cytoplasm, moderately pleomorphic round to avoid vesicular nuclei with prominent nucleoli, and two mitotic figures per high-power field. There were extensive areas of hemorrhage and necrosis along with foci of dystrophic calcification. Tumour cells were seen in the pelvis of the kidney and in a vascular channel. These are in keeping with right clear cell RCC with vascular invasion; Fuhrman's grade 3.

The woman has been regular at clinic followup. She no longer has flank pain, and pedal edema had resolved.

Postoperative ultrasound demonstrated absent right kidney, normal left kidney, no para-aortic lymphadenopathy and no ascites.

## 3. Case  2

### 3.1. Clinical Presentation

This twenty-two-year-old student was referred with 8-month history of painless total hematuria associated with dry cough, weight loss, and intermittent fever, no night sweat. He had left closed thoracostomy tube drainage done from referring center on account of left pleural effusion two weeks previously.

On physical examination, he was chronically ill looking, febrile, but with no pedal edema. He had left supraclavicular lymphadenopathy and multiple cutaneous nodules. Chest examination revealed a transverse scar in left save triangle. Abdomen showed fullness on the left flank. There was a nontender, ballotable left renal mass that extended 1 cm short of midline.

**Figure 3:**
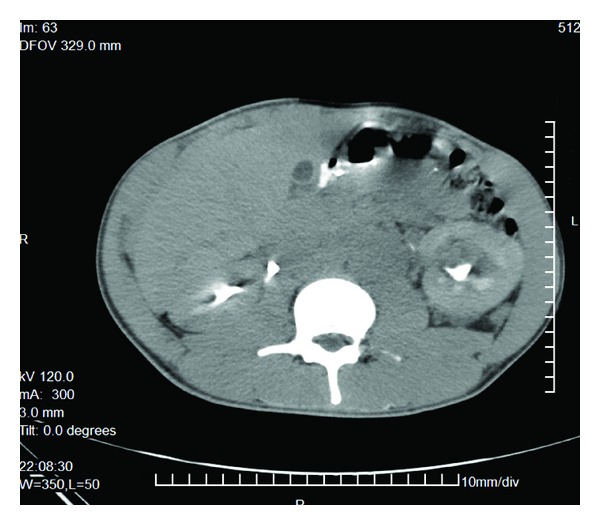


Abdominal computerized tomography revealed an enlarged left kidney. The entire parenchyma was replaced by solid mass with mixed density. There were multiple metastatic deposits on the liver (Figure 3). 

### 3.2. Operative Procedure

The peritoneal cavity was entered through a left transverse upper abdominal incision. An irregular renal mass measuring 20 cm × 11 cm was found. The left renal vein was tumor laden. There were metastatic deposits on the liver and descending colonic wall. The descending colon was reflected medially through a Todt line incision.

 The mass was mobilized inferiorly, laterally, and superiorly. The left ureter was ligated and divided as far as accessible. The renal pedicle was carefully, dissected and divided in between Satinsky clamps. The tumor, the adrenal gland, and regional lymph node were removed *en bloc*. Prerenal and postrenal IVC vascular clamps were applied, and tongue of tumour thrombus which extended from the left renal vein into vena cava was removed. Hemostasis was secured, and wound was closed in layers.

### 3.3. Outcome and Followup

The macroscopic examination of the specimen depicted a greyish-brown and dark brown kidney, with the suprarenal mass. It measured 170 cm × 110 cm × 9.5 cm and weighed 455 grams. A short ureter was demonstrated but no renal vasculature identifiable.

**Figure 4:**
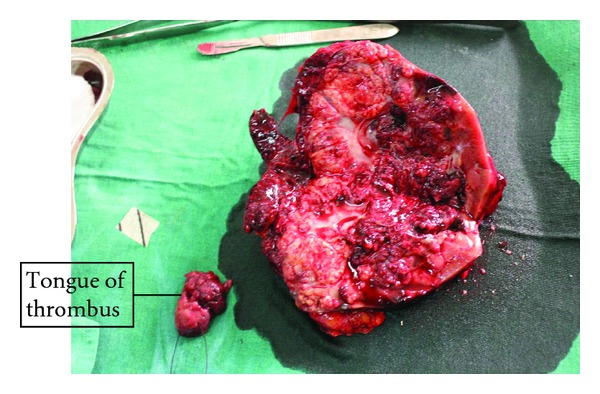


 There were accompanying dark brown and yellowish, fibrofatty, soft to firm tissue measuring 8.0 cm × 4.5 cm × 1.0 cm and weighing 16.0 grams and piece of a tagged, greyish brown, and firm tissue 4.0 cm × 3.5 cm × 2.5 cm and weighed 12.0 grams (Figure 4).

Microscopy showed a partially encapsulated lesion adjacent to hemorrhagic and edematous renal tissue. The lesion consisted of numerous papillae lined by single pseudostratified tumour cells with abundant eosinophilic cytoplasm. The nuclei were pleomorphic and vesicular, and some had prominent nucleoli. Accompanying lymph nodes showed tumor cells. A histologic diagnosis of papillary renal cell carcinoma type 2 was made. 

At six-month followup, he was found to have lost some weight but no longer has hematuria.

## 4. Case  3

### 4.1. Clinical Presentation

A forty-one-year-old nulliparous woman presented with recurrent right flank pain of 2-year duration. There was associated weight loss but no hematuria. She had appendectomy and myomectomy done two years and a year ago, respectively.

Physical examination revealed a middle-aged woman, not pale, anicteric, and with no pedal edema. Abdomen showed Pfannenstiel and right iliac fossa oblique scars with fullness on the right flank. There was nontender, palpable but not ballotable right flank mass. It extended to 14 cm below the right costal margin along the midclavicular line.

**Figure 5:**
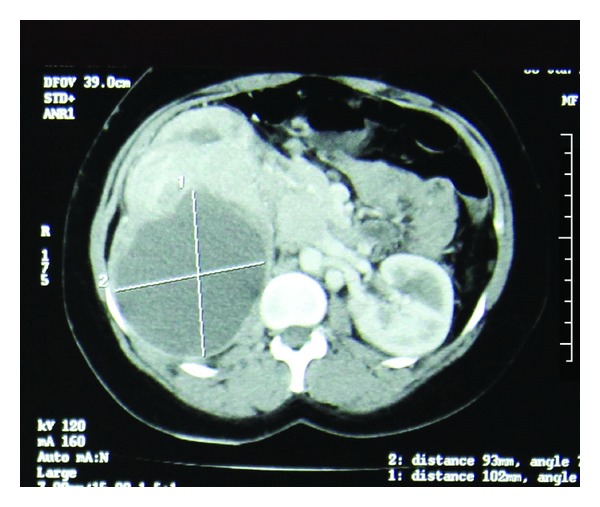


CT abdomen demonstrated an enlarged renal mass with cystic and solid components. There was tumour thrombus in the right renal vein and IVC. There was para-aortic lymphadenopathy (Figure 5). 

### 4.2. Operative Procedure

Through a right transverse upper abdominal access, the peritoneal cavity was entered.

 Dilated anterior abdominal veins were encountered and controlled. The abdomen was explored. A huge renal mass measuring 21 cm × 14 cm with mixed consistency was found. It was fixed to posterior abdominal wall. The renal vein as well as adjacent IVC was tumour laden.

There was no metastatic deposit on the liver, duodenum, and ascending colon.

The right lateral peritoneal reflection was incised to reflect the ascending colon medially. When it proved difficult to access the right renal pedicle, the mass was mobilized inferiorly, laterally, and superiorly. This maneuver enhanced the separation of the mass from the adherent psoas muscle by careful blunt dissection. The right ureter was ligated and divided far beyond the level of tumour involvement. Then, the renal vessels were secured and divided inbetween Satinsky clamps. The tumour, the adrenal, and regional lymph nodes were removed *en bloc*.

IVC thrombus was carefully assessed and venous clamps placed above the suprarenal and infrarenal vena cava thrombus levels. The opposite renal vein was gently secured, cavotomy incision made, and part of tumour thrombus removed. The cavotomy was closed with continuous 3–0 prolene. There was no drop in blood pressure during the IVC management. This could be due to extensive venous collaterals evidenced by dilated anterior abdominal wall veins. Haemostasis was secured and wound closed in layers.

She had tramadol-and metronidazole-induced emesis postoperatively. This symptom resolved upon discontinuation of the medications.

The patient was discharged to the clinic 16 days after surgery.

### 4.3. Outcome and Followup

Macroscopically, the specimen consisted of a partially incised globular mass measuring 15 cm×

14 cm × 10 cm and weighing 516 grams. The surface was light brownish and bumpy with diffuse nodules. Cut section revealed a large unilocular cyst with round grayish lining and wall thickness of approximately 0.3 cm. The adjacent area was solid, with extensive interspersed necrotic area (Figure 6).

Histologic sections of the kidney showed sheets of malignant cells which were multifocal with predominant features of papillary pattern. The perinephric fats were infiltrated by the tumour cells. These findings depicted papillary RCC with invasion to the vessels and surrounding adipose tissues.

At 3-week postoperative clinic visit, she no longer had flank pain and there was no pedal edema.

## 5. Discussion

The reviewed cases typify the common modes of representationof and care offered to advanced RCC patients in our institution. This pattern is surprisingly similar to what was reported in Lagos thirty-three years ago [3]. 

Renal cancers with caval thrombus is one of the greatest challenges for urologists because of its complex surgical management [4]. For a successful surgery, the most important factor to be considered is adequate IVC management [5].

 Among the Caucasians, RCC has its peak incidence at 4th–6th decade (mean age approximately 66 years) and prevails in male (M : F = 2 : 1). Studies from our environment have shown that RCC in Nigeria occurs in patients who are one to two decades younger than in Caucasian population [6] as Section 3 depicted and is commoner in females.

The initial clinical findings comprise haematuria (60%), flank pain (40%), and palpable flank mass (30–40%). Fever, weight loss, anemia, varicocele, paraneoplastic syndrome (5%) characterized by erythrocytosis, hypercalcemia, liver dysfunction, and amyloidosis may be found [7]. The classic triad of advanced disease comprising abdominal mass, haematuria and, pain is present in over 70% of the cases and usually in more advanced stages with poor prognosis. This contrasts sharply with what is obtained in Europe and America where only 10% of patients present late. The most common sites of metastasis are as follows: lungs (50%), bones (33%), liver (8%), cutaneous (11%), and brain (3%) [8–10].

One of the characteristics of renal carcinoma is its venotropism. Tumour thrombus may be found in the renal vein at diagnosis in 20%–35% of cases, while extension to the inferior vena cava (IVC) is seen in 4%–10% of patients [11]. The thrombus may extend above the suprahepatic vena cava up to the right atrium. When this occurs, surgery provides the only curative solution, but it is technically challenging and requires a wide understanding of the regional anatomy. The diagnosis is established through ultrasonography, and CT of abdomen in addition to chest X-rays, urinalysis, and urine cytology. Magnetic resonance imaging is indicated when involvement of the vena cava is suspected. Middleton classified four levels of cranial extension of RCC: level I, renal; level II, infrahepatic; level III, retrohepatic; level IV, atrial [12]. This classification defines thrombus location better than the TNM classification, which only differentiates between T3b when the thrombus is in the renal vein or in vena cava below the diaphragm, and T3c when the tumour extends above the diaphragm.

It is incontrovertible that palliative nephrectomy relieves intractable symptoms in the properly selected patients with metastatic disease [13], who have good performance status [14]. The pain and haematuria are aborted. Also, surgery has been shown to improve the effectiveness of adjunctive cancer therapy and quality of life of these patients as it removes the source of toxic metabolites and metastasis. In addition, regression of metastasis has been documented [15].

 Despite advances in biologic therapy for RCC, the drugs are still not accessible and affordable for most of our patients. And, since these tumours are relatively insensitive to radiotherapy or chemotherapy, the only option left is surgical excision. Such excision should be as near total as possible with only limitation being the patient's safety [3].

 If vena cava wall is invaded, total or partial resection of its wall will be required [16]. The most important precaution is to make sure that cavotomy closure leaves an adequate vein lumen for drainage of the contralateral kidney. However, if the IVC had been occluded by tumour or emboli, there should be no significant drop in blood pressure, because of formation of adequate collateral circulation. If there is a drop in blood pressure, this is corrected by a rapid infusion of blood. The involved segment of IVC is then resected, and the proximal and distal segments are closed with 5–0 vascular suture [3, 17]. Resecting the IVC in the absence of collaterals can be associated with severe edema of lower extremities.

Usually, IVC obstruction by tumour thrombus induces venous collateralization. The extent and distribution of these collaterals depend on the obstruction location, obstruction length, acute or chronic, and whether or not obstructive lesion involves tributaries of IVC [18].

 The dominant collateral systems are arranged into two groups—the superficial and the deep collaterals. The deep group includes azygous-hemiazygous, vertebral venous plexus, gonadal, ureteral, and portal (inferior mesenteric vein) while the superficial system consists of lateral thoracic, internal thoracic, and portal (paraumbilical veins).

In the lower-level IVC occlusion, azygous-hemiazygous pathway, is of great importance. Also, abdominal wall collaterals, as observed in Section 4, may occur. Midlevel obstruction induces collateralization from portal system, perinephric and capsular drainage into the azygous-hemiazygous system. However, in the upper-level obstruction, communication between the IVC and superior vena cava develops from the portal system both deep and superficial. Besides, the vertebral plexus becomes widely dilated [19].

 Surgical approach for patients with IVC tumour thrombus depends on the level of cranial extension of RCC as defined by Neves and Zincke. A chevron incision with or without a midline abdominal cephalad T extension and liver mobilisation when the thrombus is infradiaphragmatic is favoured. However, if the thrombus extends above the diaphragm, a thoracoabdominal access or midline sternotomy combined with an abdominal incision may be required.

 Cardiopulmonary bypass has been utilised as adjunct to remove cavoatrial tumour thrombus with or without hypothermic circulatory arrest. The advantages of this include careful, controlled dissection in essentially bloodless surgical field [20]. However, nonavailability of heart-lung machine is a limiting factor in most centres of the third world.

## 6. Conclusion

Inspite of operability and resectability challenges posed by advanced RCC, surgical resection remains an integral part of its care. Worrisome, however, is the persistence of late presentation and diagnosis of the disease in our environment. For over three decades now, researchers in Lagos had reechoed the abysmal prognosis of advanced cases of RCC. There is therefore the need to repackage our strategies geared at early detection of this pathology and thus improved postoperative survival.

## Figures and Tables

**Figure 6:**